# Predicting deleterious nsSNPs: an analysis of sequence and structural attributes

**DOI:** 10.1186/1471-2105-7-217

**Published:** 2006-04-21

**Authors:** Richard J Dobson, Patricia B Munroe, Mark J Caulfield, Mansoor AS Saqi

**Affiliations:** 1 Clinical Pharmacology, The William Harvey Research Institute, Bart's and the London School of Medicine and Dentistry, Queen Mary University of London, Charterhouse Square, London EC1M 6BQ, UK; 2 Bioinformatics, Institute of Cell and Molecular Science, Bart's and the London School of Medicine and Dentistry, Queen Mary University of London, Charterhouse Square, London EC1M 6BQ, UK

## Abstract

**Background:**

There has been an explosion in the number of single nucleotide polymorphisms (SNPs) within public databases. In this study we focused on non-synonymous protein coding single nucleotide polymorphisms (nsSNPs), some associated with disease and others which are thought to be neutral. We describe the distribution of both types of nsSNPs using structural and sequence based features and assess the relative value of these attributes as predictors of function using machine learning methods. We also address the common problem of balance within machine learning methods and show the effect of imbalance on nsSNP function prediction. We show that nsSNP function prediction can be significantly improved by 100% undersampling of the majority class. The learnt rules were then applied to make predictions of function on all nsSNPs within Ensembl.

**Results:**

The measure of prediction success is greatly affected by the level of imbalance in the training dataset. We found the balanced dataset that included all attributes produced the best prediction. The performance as measured by the Matthews correlation coefficient (MCC) varied between 0.49 and 0.25 depending on the imbalance. As previously observed, the degree of sequence conservation at the nsSNP position is the single most useful attribute. In addition to conservation, structural predictions made using a balanced dataset can be of value.

**Conclusion:**

The predictions for all nsSNPs within Ensembl, based on a balanced dataset using all attributes, are available as a DAS annotation. Instructions for adding the track to Ensembl are at

## Background

Single base changes in protein coding regions of DNA which lead to changes in amino acids have the potential to effect protein structure and function. These non-synonymous single nucleotide polymorphisms (nsSNPs) have been the subject of many recent studies and a large amount of data now exists in public repositories such as dbSNP [1], HGVBASE [2] and SWISSPROT [3]. Some nsSNPs are related to a disease condition but others are not associated with any change in phenotype and are regarded as neutral. Several studies have attempted to predict the functional consequences of a nsSNP, namely whether it is disease related or neutral, based on attributes of the polymorphism. Some attributes depend only on the sequence information, for example the types of residue found at the SNP location. Structural attributes such as solvent accessibility can be chosen if the protein sequence containing the nsSNP has a known 3D structure or is highly similar to a protein sequence of known structure. As structural genomics projects gain momentum an increasingly large amount of protein 3D structural information is becoming available. Mapping nsSNPs onto the corresponding 3D structures or onto the structures of proteins which are highly similar at the sequence level immediately gives a structural context to the SNP and there are databases containing such models [4]. Previous studies have sought to identify rules by which a nsSNP could be predicted to be deleterious (affect protein function) or neutral. These have included the development of empirical rules [5,6], the use of probabalistic methods [7] and machine learning [8,9]. The datasets used have included data on known nsSNPs (Wang and Moult [5], Saunders and Baker [8], Ramenski et al [6], Bao and Cui [10]) and mutation data of bacteriophage T4 lysozyme and E coli lac repressor (Chasman and Adams [7], Krishnan and Westhead [9]). Databases of coding nsSNPs have been developed by Karchin et al [11], Cavallo and Martin [12]. Some of the rules that have emerged from these approaches suggest that the majority of disease associated nsSNPs affect protein stability [5], that they are located in surface pockets of protein structures [13] and that conservation of the residue across species is an important predictive attribute [8]. Recently Bao and Cui [10] using a large collection of nsSNPs from SWISSPROT observed that structural information is useful when little information can be obtained from homologous sequences.

In this study we considered all nsSNPs described in the SWISSPROT VARIANT web pages that could be mapped onto the Ensembl database [14], allowing us to apply Ensembl annotations to these variants. This gave a set of 16,352 nsSNPs (out of a potential 18,812) of which 10,419 were associated with disease. 4217 were labelled as being neutral and 1716 were unclassified. These disease and neutral nsSNPs were contained within 893 and 1256 proteins respectively.

We surveyed the association of a number of sequence and structural attributes of nsSNPs to see if previous trends of disease and neutrality are preserved in light of the much larger datasets now available and we also included the attribute of whether the nsSNP occurs in a protein binding site [15].

One of the problems with using the available collection of natural nsSNPs is the large difference in the number of disease associated and neutral examples. To address this problem of class imbalance we also assessed the effect of resampling and weighting on the prediction performance.

## Results

### Distribution of attributes across the normal and disease associated nsSNPs

#### Non structural features

Our dataset contains 10,419 disease associated nsSNPs and 4217 nsSNPs labelled as polymorphisms. The distribution of sequence derived attributes suggests: tryptophan (W), tyrosine (Y) and cysteine (C) in the wild and mutated residues increases the chance of the nsSNP being disease related. This has previously been noted for tryptophan and cysteine by Vitkup et al. [16]. The likelihood of the nsSNP being deleterious increases as the volume, mass and hydrophobicity difference between the wild and mutated residue increases. There appeared to be very little bias in the physicochemical properties individually towards the status of the nsSNP. As previously observed, a nsSNP is much more likely to be deleterious with increasing PSIC [6] conservation score difference [8].

##### SWISSPROT features table

In Table 2 we show the most discriminatory terms from the SWISSPROT features table, namely those where over 90% of the corresponding nsSNPs are disease related. The annotation of a nsSNP in the SWISSPROT feature table is not a good discriminator between disease and polymorphic status. In our dataset those feature table terms which are predominantly associated with disease related nsSNPs have very low counts making it difficult to generalize about their utility in predicting whether a given nsSNP is disease related.

##### KEGG pathways

Our analysis of nsSNPs that map to KEGG pathways [17] revealed that the odds ratio (*P*) of deleterious to polymorphism nsSNPs (see methods for definition) is highest for the following 4 pathways: phenylalanine, ty-rosine and tryptophan biosynthesis(15.6), methionine metabolism(15.16), carbon fixation (12.56), nucleotide sugars metabolism (12.33). Assignment to a KEGG map was not used as an attribute for machine learning prediction as this result may simply reflect that these are commonly studied pathways and the pathway was considered to be a property of the protein as opposed to the nsSNP.

##### Gene Ontology

The odds ratio is highest for the following GO [18] biological processes: anti-inflammatory response (GO:0030236), peroxisome organization and biogenesis (GO:0007031), and peroxisomal membrane transport (GO:0015919). The GO cell location categories having the highest odds ratio are peroxisomal membrane (GO:0005778), integral to peroxisomal membrane (GO:0005779) and collagen type VII (GO:0005590) categories. The molecular function categories containing the highest odds ratio are phenylalanine 4-monooxygenase activity(GO:0004505), alpha-galactosidase activity (GO:0004557) and pyruvate kinase activity (GO:0004743). GO categories were not used as machine learning attributes as they were considered to be properties of the protein as opposed to the nsSNP.

##### Interactions

A total of 1944 SWISSPROT nsSNPs mapped to proteins that have entries in BIND [15]. A significant number of disease nsSNPs are within interacting regions (*x*^2 ^= 32.85, *p *= 0.001) within BIND. Table 3 shows 71.7% (odds ratio 1.29) of positions containing one or more nsSNPs that map to interacting regions are associated with disease (736 sites) as opposed to 28.3% (290 sites) which contain polymorphism nsSNPs.

#### Structural features

A total of 3821 nsSNPs could be mapped to a homologous protein of known structure. We found that of the nsSNPs that could be mapped to structure, disease nsSNPs tend to be buried and neutral nsSNPs tend to be exposed. There is also a propensity towards disease for nsSNPs occurring in beta sheets as previously noted [19] and a trend towards neutrality with increased accessibility.

##### Interactions

A total of 3028 SWISSPROT nsSNPs mapped to proteins that have structures or structural homologs in MMDBBIND [15]. Table 3 shows 86% (odds ratio 1.29) of positions containing one or more nsSNPs that map to interacting residues are associated with disease (294 sites) but also that 82% (odds ratio 0.97) of positions containing one or more nsSNPs that map to non-interacting residues are associated with disease. The difference between interacting sites containing disease nsSNPs and non-interacting sites containing disease nsSNPs is not significant (*x*^2 ^= 3.17).

All attributes excluding the KEGG pathway and GO attributes were used in further machine learning analysis.

### Machine Learning

#### Single attribute analysis

The 1R algorithm identified the best single attribute in terms of predicting disease status [20]. The attributes were ranked in terms of effectiveness as a predictor and were also ranked in terms of the information gain that they provide (Tables 4 and 5). The PSIC conservation score was identified as the best classifier in a balanced dataset achieving 72% correctly classified instances with the rules that defined a nsSNP as being disease status with a conservation score difference > 0.89 and neutral with a conservation score difference < = 0.89. These classifiers compared favourably with the conservation score rules identified by Sunyaev et al in their study [6] whereby a PSIC score difference < = 0.5 was classified as benign, 1.5 to 2.0 possibly damaging and > = 2.0 probably damaging.

#### Attribute set analysis

The J48 decision tree algorithm [20] was used to evaluate the predictive performance of the following subsets of attributes: (1)All variables. (2)Structural variables. (3)Non structurally dependant variables. (4)Non structurally dependant variables excluding the conservation score (PSIC). (5)Conservation score alone.

##### Effect of Imbalance

Attribute sets (1) and (2) contained 3821 nsSNPs when imbalanced and 1218 when balanced (see methods). Both sets included structural variables. Datasets (3), (4) and (5) contained 14,636 nsSNPs when imbalanced and 8434 when balanced. They contained more nsSNPs than sets (1) and (2) because they were not dependant on structure.

**Figure 1:**
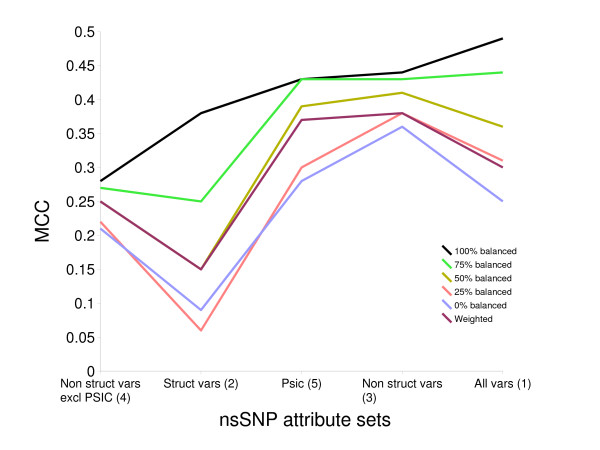
**Matthews Correlation Coeffecient (MCC) measure of predictive quality for five attribute subsets**. Set 1 – All variables (3821 nsSNPs). Set 2 – Structurally dependant variables (3821 nsSNPs). Set 3 – All non structurally dependant attributes (14.636 nsSNPs). Set 4 – Non structurally dependant variables excluding the conservation score (14.636 nsSNPs). Set 5 – The conservation score alone (14.636 nsSNPs).

The Matthews Correlation Coeffecient (MCC) increased with increasing balance within each of the sets of attributes. There was a difference in the MCC score between 0% balanced and 100% balanced of 0.24 for dataset (1), 0.29 for (2), 0.08 for (3), 0.07 for (4) and 0.15 for (5). The performance of the weighted sets lay between the level of 25% and 50% balancing for each attribute set (Figure 1).

The 100% balanced dataset (1) achieved a MCC of 0.49. When weighted and imbalanced the MCC was 0.3 and 0.25 respectively for this same set. The balanced dataset (3) was equal second in the rankings with 75% balanced (1), performing better than dataset (2). The conservation score alone achieved a similar MCC score when considered separately (MCC 0.43) as it did when it was included in dataset (3) (MCC 0.44) when 100% balanced. When the conservation score is excluded there is a drop of 0.16 in the MCC of the 100% balanced dataset (3). When set (2) is balanced it performs better than (4) but when it is not 100% balanced it has a lower MCC. Dataset (3) actually performs better than the dataset (1) when the datasets are < = 50% balanced or weighted. The imbalanced dataset (2) achieved the lowest MCC score.

The rules learnt from the machine learning approach were applied to make predictions on nsSNPs where the nsSNP status was unknown. All nsSNPs within Ensembl (Build 27_1) were used as the unknown test dataset. The dataset was trained on the 100% balanced dataset of 609 neutral and 609 disease nsSNPs using all variables. This resulted in a predicted classification along with a confidence score for each of the 'unseen' nsSNPs with Ensembl.

## Discussion

The use of a 100% balanced dataset dramatically increases the Matthews correlation coefficient (MCC) and removes any bias towards building rules for prediction of the disease state. Complete undersampling is a better choice than reweighting in addressing an imbalanced dataset. When imbalanced, performance using conservation alone (MCC 0.28) is close to that achieved by Bao and Cui [10] (MCC 0.305) yet with a balanced dataset the MCC is greatly improved (MCC 0.43).

We see a larger spread in the MCC when using the smaller datasets that include structural variables because of the larger ratio of disease to neutral nsSNPs in these datasets. This explains why the performance for the dataset of all variables (as measured by MCC) is good when >50% balanced, yet drops below that of non structurally dependant variables when the level of balance falls below this figure. It also explains the similar pattern seen when comparing structurally dependant variables and non structurally dependant variables excluding conservation, except that the cut off lies at the 75% level of balance.

There are a number of caveats with the training dataset. The dataset may include nsSNPs predicted to be 'disease' where some of the nsSNPs may only be in linkage disequilibrium with the phenotype in question and may themselves not be causative. This 'pollutes' the training set and may lead to a higher error rate and lower MCC. Further filtering of the dataset would lead to a smaller but cleaner training set that would in turn lead to lower error rates and an increase in the MCC. Further complications could arise where molecular phenotypic changes that don't result in a physical phenotype and unstudied or unobserved phenotypic changes may result in a nsSNP being classified as neutral that should be classified as disease. Improvements to the system could also be made if SNPs could be graded in terms of how damaging they are as opposed to the boolean states of disease and polymorphism that currently classifies them. In time databases may contain this information.

## Conclusion

Reassuringly, previously observed trends can be seen in this study of a large number of nsSNPs. Disease nsSNPs tend to affect protein stability [5], are buried [13] and disruption of a conserved residue is an important predictive attribute [8]. We extend previous work by addressing the problems of imbalance and redundancy within the attributes for a large selection of natural nsSNPs and then go on to make predictions on all Ensembl nsSNPs. Bao and Cui [10] and Saunders and Baker [8] showed that in the absence of a conservation score, structural attributes are valuable predictors. Here we affirm using machine learning methods that the sequence conservation measure is the most powerful single predictor and we are able to show that a high level of accuracy is achieved using the conservation score alone. We are also able to show that structural attributes in combination with the conservation score improves prediction accuracy but also that there are other non structurally dependant attributes that can reduce the error rate further and are valuable in the absence of a conservation score. The performance of all attribute subsets however, is very much dependant on how the datasets are configured. The maximum prediction accuracy can be achieved by combining all attributes of the nsSNP within a balanced dataset.

## Methods

### SNP Dataset

The SWISSPROT VARIANT web pages [4] provide information on single amino acid polymorphisms associated with a given SWISSPROT entry. The variants are labelled as disease, unclassified or polymorphism. A subset of these SNPs were used in this study, namely those where the amino acid polymorphism was found to map onto the Ensembl human genome protein sequence. A mapped SNP was kept where the amino acid was the same in both the SWISSPROT sequence and the Ensembl protein sequence and the aligned region had an E value < 1*e *- 10 over a region > 100 amino acids in length. This gave a set of 16,352 variants which mapped to Ensembl of which 10,419 were related to disease (64%), 4217 (26%) were labelled as a polymorphism and the rest were unclassified. Matches to known structure and to structural homologs were obtained in the following way: each sequence containing a nsSNP was searched against all the sequences in the protein data bank (PDB) using the PSIBLAST program [21] with ten iterations. Only hits with an E value of less than 1e-10 where the amino acids at the position of the nsSNP were the same were stored. Each of these nsSNP containing SWISSPROT entries was aligned with the sequence in a relevant HSSP [22] file (database of homology-derived secondary structure of proteins). Where there were multiple PDB annotations in the SWISSPROT file, the PDB with the lowest E value was used. A total of 500 nsSNP-containing proteins had structural homologs, of which 299 proteins contained disease related nsSNPs and 295 contained polymorphic nsSNPs (a protein can contain both disease and polymorphic nsSNPS). The data is summarised in Table 1.

### nsSNP Features

As the subset of nsSNP containing proteins with associated 3D structures is considerably smaller than the set of all nsSNP containing proteins we considered the set of structurally dependant features separately from the set of features that were not dependant on structure. A total of 17 features were used, 11 non structurally dependant and 6 structurally dependant.

#### Non structural features

The features chosen were largely based on those used by Krishnan and Westhead [9] and Ramenski et al. [6]: (1) The residue types of the original and mutated residues. (2) The physicochemical properties of the original and mutated residues. (3) Sequence conservation: is the nsSNP at a conserved position. The sequence was matched against a protein non redundant database using the BLAST program and all hits with an E value less than 0.0005 were stored. A multiple alignment was constructed and sequence variation at the position of the nsSNP was described by calculating the PSIC (position-specific counts of independent observations) score [6]. (4) PAM (accepted point mutations) score shift measured from the PAM120 matrix [23]. (5) Side chain volume change [24]. (6) Mass change. The molecular weights are those of the neutral, free amino acids. (7) Hydrophobicity difference [25]. Four further non structurally dependant attributes were taken from the SWISSPROT features table, pathway information, ontology classifications and interacting regions.

##### SWISSPROT features table

The SWISSPROT entry feature table may contain information about functional sites. A survey was carried out of functional site terms across all nsSNPs in the SWISSPROT VARIANT pages. Following Ramensky et al [6], nsSNPs landing within the following labelled features: ACT_SITE, BINDING, MOD_RES, SITE, LIPID, METAL, DISULFID, CROSSLNK, TRANSMEM, SIGNAL, PROPEP were considered to be termed 'functional' sites for the benefit of the machine learning analysis. For each labelled feature, *i*, we calculated the odds ratio *P*_*i*_:

Pi=Ndisi/NpolyiNdistot/Npolytot
 MathType@MTEF@5@5@+=feaafiart1ev1aaatCvAUfKttLearuWrP9MDH5MBPbIqV92AaeXatLxBI9gBaebbnrfifHhDYfgasaacH8akY=wiFfYdH8Gipec8Eeeu0xXdbba9frFj0=OqFfea0dXdd9vqai=hGuQ8kuc9pgc9s8qqaq=dirpe0xb9q8qiLsFr0=vr0=vr0dc8meaabaqaciaacaGaaeqabaqabeGadaaakeaacqWGqbaudaWgaaWcbaGaemyAaKgabeaakiabg2da9maalaaabaGaemOta40aa0baaSqaaiabdsgaKjabdMgaPjabdohaZbqaaiabdMgaPbaakiabc+caViabd6eaonaaDaaaleaacqWGWbaCcqWGVbWBcqWGSbaBcqWG5bqEaeaacqWGPbqAaaaakeaacqWGobGtdaqhaaWcbaGaemizaqMaemyAaKMaem4CamhabaGaemiDaqNaem4Ba8MaemiDaqhaaOGaei4la8IaemOta40aa0baaSqaaiabdchaWjabd+gaVjabdYgaSjabdMha5bqaaiabdsha0jabd+gaVjabdsha0baaaaaaaa@5684@

where Ndisi
 MathType@MTEF@5@5@+=feaafiart1ev1aaatCvAUfKttLearuWrP9MDH5MBPbIqV92AaeXatLxBI9gBaebbnrfifHhDYfgasaacH8akY=wiFfYdH8Gipec8Eeeu0xXdbba9frFj0=OqFfea0dXdd9vqai=hGuQ8kuc9pgc9s8qqaq=dirpe0xb9q8qiLsFr0=vr0=vr0dc8meaabaqaciaacaGaaeqabaqabeGadaaakeaacqWGobGtdaqhaaWcbaGaemizaqMaemyAaKMaem4CamhabaGaemyAaKgaaaaa@3374@ is the number of disease nsSNPs in the feature *i *and Ndistot
 MathType@MTEF@5@5@+=feaafiart1ev1aaatCvAUfKttLearuWrP9MDH5MBPbIqV92AaeXatLxBI9gBaebbnrfifHhDYfgasaacH8akY=wiFfYdH8Gipec8Eeeu0xXdbba9frFj0=OqFfea0dXdd9vqai=hGuQ8kuc9pgc9s8qqaq=dirpe0xb9q8qiLsFr0=vr0=vr0dc8meaabaqaciaacaGaaeqabaqabeGadaaakeaacqWGobGtdaqhaaWcbaGaemizaqMaemyAaKMaem4CamhabaGaemiDaqNaem4Ba8MaemiDaqhaaaaa@3662@ is the total number of disease nsSNPS in our dataset and similarly for polymorphic nsSNPs.

##### KEGG pathways

In order to observe the distribution of disease and neutral nsSNPs within pathways we mapped the set of 16,352 nsSNPs to KEGG pathways [17]. For each pathway, *i*, we calculated the odds ratio *P*_*i *_where Ndisi
 MathType@MTEF@5@5@+=feaafiart1ev1aaatCvAUfKttLearuWrP9MDH5MBPbIqV92AaeXatLxBI9gBaebbnrfifHhDYfgasaacH8akY=wiFfYdH8Gipec8Eeeu0xXdbba9frFj0=OqFfea0dXdd9vqai=hGuQ8kuc9pgc9s8qqaq=dirpe0xb9q8qiLsFr0=vr0=vr0dc8meaabaqaciaacaGaaeqabaqabeGadaaakeaacqWGobGtdaqhaaWcbaGaemizaqMaemyAaKMaem4CamhabaGaemyAaKgaaaaa@3374@ is the number of disease nsSNPs in pathway *i *and Ndistot
 MathType@MTEF@5@5@+=feaafiart1ev1aaatCvAUfKttLearuWrP9MDH5MBPbIqV92AaeXatLxBI9gBaebbnrfifHhDYfgasaacH8akY=wiFfYdH8Gipec8Eeeu0xXdbba9frFj0=OqFfea0dXdd9vqai=hGuQ8kuc9pgc9s8qqaq=dirpe0xb9q8qiLsFr0=vr0=vr0dc8meaabaqaciaacaGaaeqabaqabeGadaaakeaacqWGobGtdaqhaaWcbaGaemizaqMaemyAaKMaem4CamhabaGaemiDaqNaem4Ba8MaemiDaqhaaaaa@3662@ is the total number of disease nsSNPS in our dataset and similarly for polymorphic nsSNPs.

##### Gene Ontology

Each nsSNP containing protein sequence belongs to a number of Gene Ontology (GO) categories [18]. The odds ratio of neutral and disease nsSNPs were calculated for each of the GO categories.

##### Interactions

The BIND [15] database was used to map nsSNPs to interacting regions. A potential interacting region was defined as a region from amino acid position n to amino acid position m. These interactions were generally regions observed experimentally and were not considered structurally dependant annotations as the BIND database entries have sequence identifiers. The odds ratio *P*_*i *_was calculated where Ndisi
 MathType@MTEF@5@5@+=feaafiart1ev1aaatCvAUfKttLearuWrP9MDH5MBPbIqV92AaeXatLxBI9gBaebbnrfifHhDYfgasaacH8akY=wiFfYdH8Gipec8Eeeu0xXdbba9frFj0=OqFfea0dXdd9vqai=hGuQ8kuc9pgc9s8qqaq=dirpe0xb9q8qiLsFr0=vr0=vr0dc8meaabaqaciaacaGaaeqabaqabeGadaaakeaacqWGobGtdaqhaaWcbaGaemizaqMaemyAaKMaem4CamhabaGaemyAaKgaaaaa@3374@ is the number of sites containing disease nsSNPs in either an interacting region or non-interacting region *i *and Ndistot
 MathType@MTEF@5@5@+=feaafiart1ev1aaatCvAUfKttLearuWrP9MDH5MBPbIqV92AaeXatLxBI9gBaebbnrfifHhDYfgasaacH8akY=wiFfYdH8Gipec8Eeeu0xXdbba9frFj0=OqFfea0dXdd9vqai=hGuQ8kuc9pgc9s8qqaq=dirpe0xb9q8qiLsFr0=vr0=vr0dc8meaabaqaciaacaGaaeqabaqabeGadaaakeaacqWGobGtdaqhaaWcbaGaemizaqMaemyAaKMaem4CamhabaGaemiDaqNaem4Ba8MaemiDaqhaaaaa@3662@ is the total number of sites containing disease nsSNPS in our dataset that map to BIND and similarly for polymorphic nsSNPs.

#### Structural features

The following structural attributes were extracted from the corresponding HSSP file [22]: (1) Secondary structure conformation: residue in is isolated beta-bridge (single pair beta-sheet hydrogen bond formation), 5 turn helix (pi helix), 3 turn helix (3/10 helix), 4 turn helix (alpha helix), bend, beta sheet in parallel and/or anti-parallel sheet conformation (extended strand), hydrogen bonded turn (3. 4 or 5 turn). (2) Relative solvent accessibility. (3) Normalised relative accessibility. (4) Exposure (relative accessibility as 3 states). (5) Buried charge.

Relative accessibility and normalised relative accessibility were calculated in the same manner as Chasman and Adams [7]. The maximum accessible surface area (Å^2^) reference values are those calculated for residues in a Gly-Xaa-Gly tripeptide in extended conformation [26]. In order to group the relative accessibility, it was projected onto 3 states: buried (here defined as <9% relative accessibility), intermediate (9% ≤ rel. acc. < 36%), exposed (rel. acc. ≥ 36%) [27]. Buried charge is defined as K.R.D.E.H wild type amino acid and 'buried' exposure class. [9]

##### Interactions

The MMDBBIND database [15] was used as a second source to map nsSNPs to interacting regions. MMDBBIND contains atomic level details of interactions. These interactions are annotated automatically from MMDB [28] which is a subset of experimentally determined PDB structures. This attribute is therefore dependant on structure as it requires a PDB identifier. MMDBBIND interactions are a much more precise interaction annotation than the BIND interactions as the BIND defined regions can sometimes be very large in amino acid length. Again, the odds ratio *P*_*i *_was calculated where Ndisi
 MathType@MTEF@5@5@+=feaafiart1ev1aaatCvAUfKttLearuWrP9MDH5MBPbIqV92AaeXatLxBI9gBaebbnrfifHhDYfgasaacH8akY=wiFfYdH8Gipec8Eeeu0xXdbba9frFj0=OqFfea0dXdd9vqai=hGuQ8kuc9pgc9s8qqaq=dirpe0xb9q8qiLsFr0=vr0=vr0dc8meaabaqaciaacaGaaeqabaqabeGadaaakeaacqWGobGtdaqhaaWcbaGaemizaqMaemyAaKMaem4CamhabaGaemyAaKgaaaaa@3374@ is the number of sites containing disease nsSNPs in either an interacting region or non-interacting region *i *and Ndistot
 MathType@MTEF@5@5@+=feaafiart1ev1aaatCvAUfKttLearuWrP9MDH5MBPbIqV92AaeXatLxBI9gBaebbnrfifHhDYfgasaacH8akY=wiFfYdH8Gipec8Eeeu0xXdbba9frFj0=OqFfea0dXdd9vqai=hGuQ8kuc9pgc9s8qqaq=dirpe0xb9q8qiLsFr0=vr0=vr0dc8meaabaqaciaacaGaaeqabaqabeGadaaakeaacqWGobGtdaqhaaWcbaGaemizaqMaemyAaKMaem4CamhabaGaemiDaqNaem4Ba8MaemiDaqhaaaaa@3662@ is the total number of sites containing disease nsSNPS in our dataset that map to MMDBBIND and similarly for polymorphic nsSNPs.

### Machine Learning

All machine learning analysis was performed using the *WEKA *package of machine learning algorithms [20].

#### Single attribute analysis

In order to identify the most effective classifier from all of the attributes, the 1R classifying algorithm, which uses the minimum-error attribute for prediction, was used with a minimum bucket size of 14 and 10 fold cross validation on the fully balanced dataset containing all variables. The bucket size of 14 was chosen because bucket sizes below this value caused overfitting and/or an increase in the error rate. The attributes were then ranked in terms of their effectiveness as a predictor using the default ranker search method with this 1R attribute evaluator and were also ranked in terms of the information gain that they provide [20]. Entropy is a measure of information and represents the amount of information that would still be needed to classify the nsSNP having used the attribute in question [29]. The information gain is the information required after using the attribute as a classifier subtracted from the information required before using the attribute as a classifier.

#### Attribute set analysis

It is of value to investigate the relative importance of attributes that require structure and those that can be obtained by sequence alone. The importance of sequence conservation has been previously noted [8] so it was also important to observe whether the other non structurally dependant attributes could add to prediction quality achieved with conservation score alone. Hence, we compared predictions for the following sets of selected attributes:

(1) All variables (3821 nsSNPs). (2) Structurally dependant variables (3821 nsSNPs). (3) All non structurally dependant attributes (14,636 nsSNPs). (4) Non structurally dependant variables excluding the conservation score (14,636 nsSNPs). (5) The conservation score alone (14,636 nsSNPs).

Decision trees have been shown to perform well in a mixed cross   validated training dataset [9]. They also provide a confidence score and intelligible rules to a prediction. Based on this knowledge we decided to use the J48 decision tree classifier to analyze the assembled sets of variables. J48 was run with the default set of parameters and 10 fold cross validation.

##### Effects of Imbalance

There was a problem of imbalance [30] within the dataset which introduced skewing towards the avoidance of errors for the disease status as there are 2.5 times more disease nsSNPs than neutral. The imbalanced dataset applies a higher cost to getting a disease prediction wrong, meaning that the rules inferred by the imbalanced dataset are able to predict disease status but unable to predict neutral nsSNPs accurately. In fact, in this instance the algorithm makes more incorrect neutral predictions than correct ones. The effect of imbalance depends on total set size, class heterogeneity, data complexity and the classification technique. To address this problem of imbalance we applied cost-sensitive classification by either resampling or reweighting [20]. Resampling can be used to either increase the number of the minority class (*oversample*) or reduce the number in the majority class (*undersample*) [31]. Reweighting can be used to apply a cost to an incorrectly classified minority class without altering the numbers in each class. The cost is directly proportional to the imbalance. Here we compare results obtained by using both resampling and reweighting. We undersampled the disease class as oversampling would make exact copies of the neutral class, potentially resulting in overfitting of the data. Undersampling results in the loss of information so it was decided to randomly undersample at rates of 100%, 75%, 50%, 25% and 0%. This means that at each rate, '*n*% of the excess members of the majority class were randomly removed' [30], resulting in a balanced dataset when undersampling at a rate of 100%.

##### Attribute redundancy

Some attributes may work well in combination leaving other attributes redundant and maybe even causing a reduction in prediction quality. The optimised subset of attributes for each attribute set at each level of imbalance was obtained using wrapper-based feature selection with J48 as the learning method with default option settings. The wrapper-based feature selection method in combination with the Genetic Search algorithm produced the lowest error rates [20]. The Genetic Search algorithm was initialised with a population size of 20 and then 50 generations were evaluated.

##### Measure of prediction quality

Matthews correlation coefficient (MCC) is a more appropriate measure of prediction performance than the error rate (E)

E=FP+FNTP+TN+FP+FN
 MathType@MTEF@5@5@+=feaafiart1ev1aaatCvAUfKttLearuWrP9MDH5MBPbIqV92AaeXatLxBI9gBaebbnrfifHhDYfgasaacH8akY=wiFfYdH8Gipec8Eeeu0xXdbba9frFj0=OqFfea0dXdd9vqai=hGuQ8kuc9pgc9s8qqaq=dirpe0xb9q8qiLsFr0=vr0=vr0dc8meaabaqaciaacaGaaeqabaqabeGadaaakeaacqWGfbqrcqGH9aqpdaWcaaqaaiabdAeagjabdcfaqjabgUcaRiabdAeagjabd6eaobqaaiabdsfaujabdcfaqjabgUcaRiabdsfaujabd6eaojabgUcaRiabdAeagjabdcfaqjabgUcaRiabdAeagjabd6eaobaaaaa@3FFD@

because in a case where all samples are assigned to a majority class, E may still be low [32]. Matthews correlation coefficient combines both sensitivity and specificity into one measure and lies in the range -1 to 1 with 1 meaning complete prediction accuracy, 0 meaning every prediction was randomly assigned. MCC is defined by

MCC=(TP.TN−FP.FN)(TN+FN)(TN+FP)(TP+FN)(TP+FP)
 MathType@MTEF@5@5@+=feaafiart1ev1aaatCvAUfKttLearuWrP9MDH5MBPbIqV92AaeXatLxBI9gBaebbnrfifHhDYfgasaacH8akY=wiFfYdH8Gipec8Eeeu0xXdbba9frFj0=OqFfea0dXdd9vqai=hGuQ8kuc9pgc9s8qqaq=dirpe0xb9q8qiLsFr0=vr0=vr0dc8meaabaqaciaacaGaaeqabaqabeGadaaakeaacqWGnbqtcqWGdbWqcqWGdbWqcqGH9aqpdaWcaaqaamaabmaabaGaemivaqLaemiuaaLaeiOla4IaemivaqLaemOta4KaeyOeI0IaemOrayKaemiuaaLaeiOla4IaemOrayKaemOta4eacaGLOaGaayzkaaaabaWaaOaaaeaadaqadaqaaiabdsfaujabd6eaojabgUcaRiabdAeagjabd6eaobGaayjkaiaawMcaamaabmaabaGaemivaqLaemOta4Kaey4kaSIaemOrayKaemiuaafacaGLOaGaayzkaaWaaeWaaeaacqWGubavcqWGqbaucqGHRaWkcqWGgbGrcqWGobGtaiaawIcacaGLPaaadaqadaqaaiabdsfaujabdcfaqjabgUcaRiabdAeagjabdcfaqbGaayjkaiaawMcaaaWcbeaaaaaaaa@5A80@

where TP is true positive, FP is false positive, TN is true negative and FN is false negative.

## Availability

The predictions of nsSNP status for all of the Ensembl nsSNPs (Build 27_1) made using the 100% balanced dataset of 609 neutral and 609 disease nsSNPs with all variables for training, are available to be viewed within Ensembl as a Distributed Annotation System (DAS) [33] source. Instructions for adding the annotation track can be found at .

## Appendix

On a suggestion of one of the referees, we have investigated how trees trained on balanced datasets perform on imbalanced datasets. The decision tree analysis was repeated except that prior to the cross validation, a random 20% of the instances within each attribute set were removed (retaining the level of imbalance in the original attribute set) for later re-evaluation of the trained model. The cross validation was then performed at the undersampled levels of 100%, 75%, 50%, 20% and 0% as before, on the remaining 80% of the atribute set. The trained model was then re-evaluated on the initially excluded imbalanced 20%.

The rankings at the differing levels of undersampling was much the same as the original cross validation, with the MCC generally increasing with increasing level of balance. Again, weighting the dataset was generally not as effective as undersampling. One point to note is that the overall performance of the smaller attribute sets(the ones that contain structural attributes) drops slightly in relation to the attribute sets not dependant on structure. This effect is probably due to the 20% reduction in size of the trained tree. This drop in sample size appears to have a greater effect on the performance of the smaller datasets that are structurally dependant.

## Authors' contributions

RJD wrote the code (except for PSIC). PBM, MASS supervised the project and provided input in the design. RJD, PBM, MASS were involved in the preparation of the manuscript. RJD, PBM, MJC, MASS read and approved the manuscript.

## Figures and Tables

**Table 1:** Summary of Training Dataset

	Disease	Polymorphism	Total
Number of nsSNPS	10,419	4217	14,636
Number of nsSNPS within proteins with structural homologs	3212	609	3821
Number of Proteins with nsSNPs	893	1256	2149
Number of Proteins with nsSNPs having structural homologs	299	295	594

**Table 2:** The number of disease and polymorphism nsSNPs within SWISSPROT feature table sites containing > 90% disease nsSNPs.

Site	Disease	Polymorphisms	Percentage (odds ratio) of nsSNPs within these sites that are disease
ACT_SITE	25	1	96.15 (10.12)
BINDING	13	0	100 (-)
DNA_BIND	352	20	94.62 (7.12)
METAL	38	0	100 (-)
MOD_RES	34	3	91.89 (4.59)
MUTAGEN	111	10	91.74 (4.49)
NP_BIND	108	8	93.1 (5.46)

**Table 3:** Distribution of positions containing disease and neutral nsSNPs within BIND and MMDBBIND. Some sites may contain multiple nsSNPs

	Interacting sites (num) [odds ratio]	Non-interacting sites (num) [odds ratio]
Disease (BIND)	71.7%(736) [1.29]	58.6%(431) [0.72]
Polymorphism (BIND)	28.3%(290)	41.4%(304)
Disease (MMDBBIND)	86.0%(294) [1.29]	82.0%(1818) [0.97]
Polymorphism (MMDBBIND)	14.0%(48)	18.0%(398)

**Table 4:** Top 10 rank of attributes using 1R with 10 fold cross validation and bucket size 14

1R Rank	Attribute
72.82	conservation score (PSIC)
67.49	norm relative accessibility
63.46	MMDBBIND
62.64	mass change
62.56	relative accessibility
62.23	exposure
61.41	PAM score
60.67	mutation residue
60.34	volume change
59.19	wild type residue

**Table 5:** Top 10 information gain attributes

Info gain (bits)	Attribute
0.2	conservation score (PSIC)
0.1	norm relative accessibility
0.09	wild type residue
0.07	relative accessibility
0.06	PAM score
0.06	mass change
0.05	mutation residue
0.05	exposure
0.04	volume change
0.04	hydrophobicity difference
